# A miRNA-based classification of renal cell carcinoma subtypes by PCR and *in situ* hybridization

**DOI:** 10.18632/oncotarget.23162

**Published:** 2017-12-08

**Authors:** Ashley Di Meo, Rola Saleeb, Samantha J. Wala, Heba W. Khella, Qiang Ding, Haiyan Zhai, Kalra Krishan, Adriana Krizova, Manal Gabril, Andrew Evans, Fadi Brimo, Maria D. Pasic, Antonio Finelli, Eleftherios P. Diamandis, George M. Yousef

**Affiliations:** ^1^ Department of Laboratory Medicine, Keenan Research Centre for Biomedical Science, Li Ka Shing Knowledge Institute, St. Michael's Hospital, Toronto, ON, Canada; ^2^ Department of Laboratory Medicine and Pathobiology, University of Toronto, Toronto, ON, Canada; ^3^ Department of Pathology and Laboratory Medicine, Mount Sinai Hospital, Toronto, ON, Canada; ^4^ BioGenex Laboratories, Fremont, CA, United States of America; ^5^ Department of Pathology, London Health Sciences Center and Western University, London, ON, Canada; ^6^ Department of Pathology, McGill University Health Centre, Montreal, QC, Canada; ^7^ Department of Laboratory Medicine, St. Joseph's Health Centre, Toronto, ON, Canada; ^8^ Division of Urologic Oncology, Princess Margaret Hospital, University Health Network, Department of Surgery, University of Toronto, Toronto, ON, Canada

**Keywords:** renal oncocytoma, miRNA, precision medicine, renal cell carcinoma, in situ hybridization

## Abstract

Renal cell carcinoma (RCC) constitutes an array of morphologically and genetically distinct tumors the most prevalent of which are clear cell, papillary, and chromophobe RCC. Accurate distinction between the typically benign-behaving renal oncocytoma and RCC subtypes is a frequent challenge for pathologists. This is critical for clinical decision making. Subtypes also have different survival outcomes and responses to therapy. We extracted RNA from ninety formalin-fixed paraffin-embedded (FFPE) tissues (27 clear cell, 29 papillary, 19 chromophobe, 4 unclassified RCC and 11 oncocytomas). We quantified the expression of six miRNAs (miR-221, miR-222, miR-126, miR-182, miR-200b and miR-200c) by qRT-PCR, and by *in situ* hybridization in an independent set of tumors. We developed a two-step classifier. In the first step, it uses expression of either miR-221 or miR-222 to distinguish the clear cell and papillary subtypes from chromophobe RCC and oncocytoma (miR-221 AUC: 0.96, 95% CI: 0.9132–1.014, *p* < 0.0001 and miR-222 AUC: 0.91, 95% CI: 0.8478–0.9772, *p* < 0.0001). In the second step, it uses miR-126 to discriminate clear cell from papillary RCC (AUC: 1, *p* < 0.0001) and miR-200b to discriminate chromophobe RCC from oncocytoma (AUC: 0.95, 95% CI: 0.8933–1.021, *p* < 0.0001). *In situ* hybridization showed a nuclear staining pattern. miR-126, miR-222 and miR-200b were significantly differentially expressed between the subtypes by *in situ* hybridization. miRNA expression could distinguish RCC subtypes and oncocytoma. miRNA expression assessed by either PCR or *in situ* hybridization can be a clinically useful diagnostic tool to complement morphologic renal tumor classification, improving diagnosis and patient management.

## INTRODUCTION

Renal cell carcinoma (RCC) is the most prevalent malignancy of the adult kidney, accounting for 90% of all kidney cancers [1, 2]. Worldwide, over 260, 000 cases of kidney cancer are diagnosed annually and up to 116, 000 people die as a result of the disease [2]. RCC comprises a heterogeneous group of tumors with distinct genetic and molecular characteristics [3]. Clear cell RCC (ccRCC), papillary RCC (pRCC) and chromophobe RCC (chRCC) are the most common RCC subtypes. In addition, _~_4–5% of RCC cases are labelled “unclassified” [4]. Oncocytoma, a benign neoplasm of the kidney, is found in up to 25% of early stage tumors managed with surgery [5].

Characterization of RCC subtypes relies on distinct histopathological characteristics. Clear cell tumors are hypervasular and usually have abundant clear cytoplasm while papillary tumors display papillary architecture with fibrovascular cores containing variable amounts of foamy macrophages. Chromophobe tumors have distinct cell borders, voluminous reticular cytoplasm, and perinuclear halos. Oncocytoma is characterized by polygonal cells that have abundant eosinophilic cytoplasm in addition to uniform rounded nuclei. However, morphology is not always conclusive and overlapping features certainly exist with tumors characterized by clear cytoplasm, tubulopapillary architecture and/or eosinophilic cytoplasm having a broad differential diagnosis. For instance, the eosinophilic variants of chRCC and ccRCC can exhibit similar cytological features. In addition, hybrid oncocytic chromophobe tumors (HOCT) contain histological features from both chromophobe RCC and renal oncocytoma. Inter-observer variability amongst pathologists has also been reported [6]. Moreover, unclassified RCC exhibits variable microscopic features that do not fit any defined subtype [7].

RCC subtypes exhibit different clinical behaviour, prognosis and response to therapy [8]. ccRCC is most likely to present at advanced stage, higher grade and metastatic disease. In fact, 20–30% of clear cell patients present with metastasis at diagnosis. Additionally, clear cell tumors have worse cancer-specific and overall survival (OS) compared to papillary and chromophobe RCC [9]. Although less aggressive, papillary RCC type II often presents at higher stage and grade [10] whereas chromophobe tumors are considered more indolent [11]. Accurate classification of RCC is also of significant clinical importance for guiding type-specific treatment strategies in the new era of targeted therapy.

More recently, molecular markers showed promise as adjuvant tools for accurate classification by complementing morphologic evaluation, especially in small biopsies where diagnostic materials can be limited [12]. Reports have shown the ability of molecular signatures to “biologically” classify RCC. For instance, the two subtypes of pRCC were shown to have distinct biological signatures [13]. miRNAs are short non-coding RNA fragments that control gene expression by pairing to the 3’UTR of target messenger RNA (mRNA). The link between miRNAs and cancer is well established in literature as they have been shown to play a key role in tumorigenesis and aggressive behaviour of tumors [14]. miRNAs have gained recognition as promising diagnostic, prognostic and predictive RCC biomarkers [15–17]. They have a number of advantages. They are highly stable and can be extracted from body fluids and formalin-fixed paraffin embedded (FFPE) tissues [18]. Earlier reports, including ours, have shown that miRNAs are differentially expressed between RCC subtypes [19–21]. We previously identified 65 miRNAs that were able to accurately differentiate between normal kidney, ccRCC, pRCC, chRCC and renal oncocytoma [19].

In this study, we measured the expression of six miRNAs in ninety FFPE renal tumor samples by qRT-PCR. We developed a miRNA classifier that is able to differentiate between ccRCC, pRCC, and chRCC as well as renal oncocytoma in two steps. miRNAs included in the classification scheme were then quantified in four cases of unclassified RCC. We also examined the diagnostic utility of our miRNAs by *in situ* hybridization in an independent set of 98 FFPE RCC tissues.

## RESULTS

### miRNA expression can classify renal cell carcinoma subtypes

**Figure 1:**
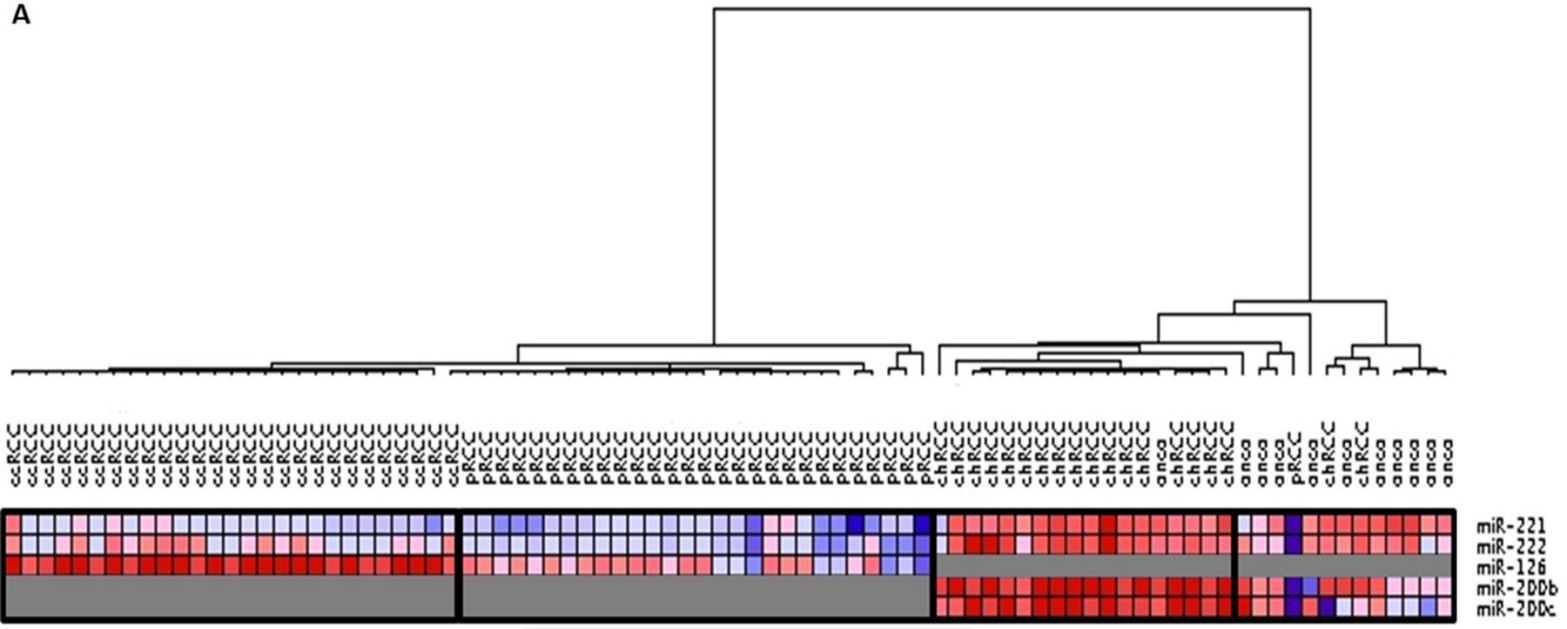

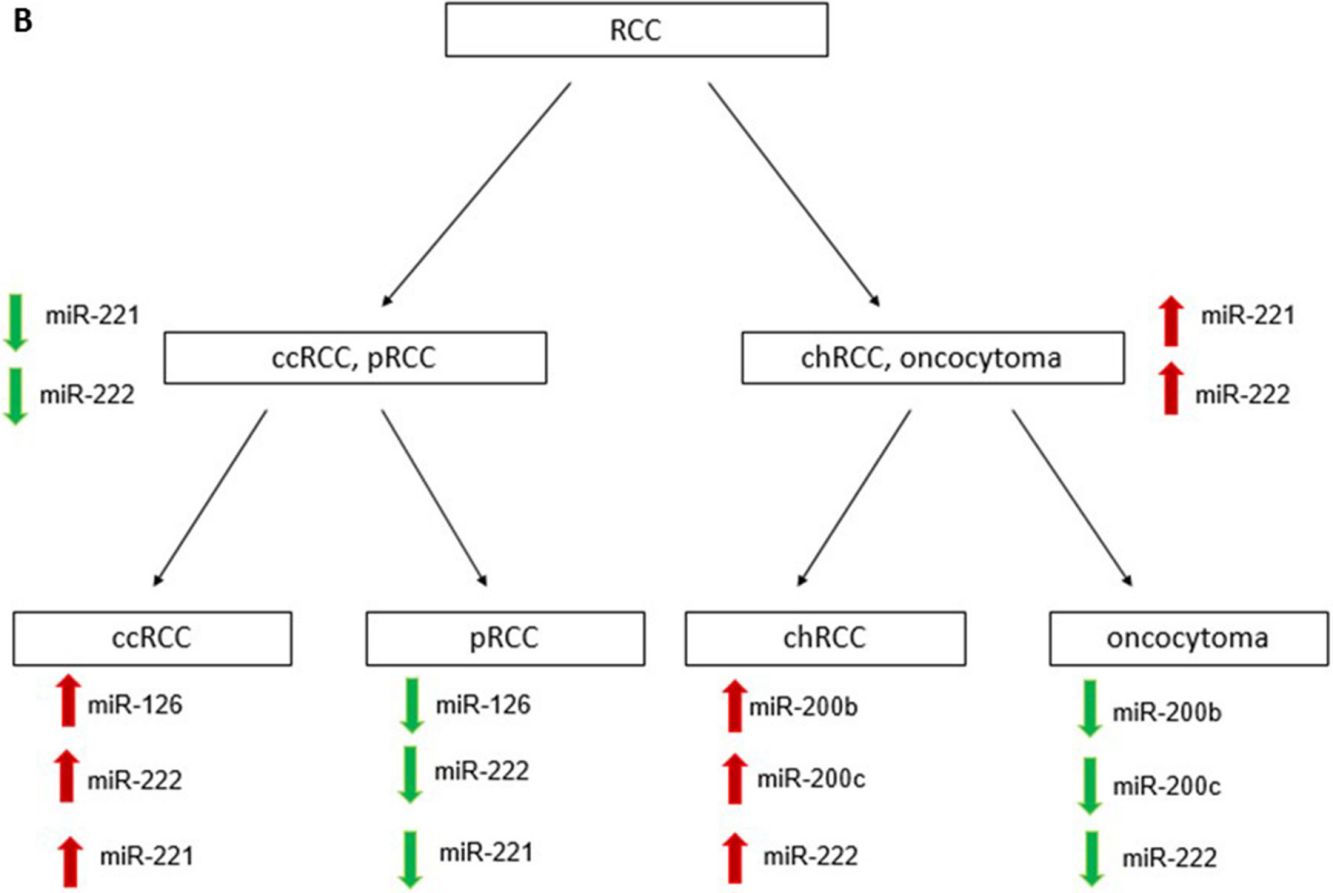
(**A**) Unsupervised hierarchical clustering of kidney tumors by miRNA expression. The samples clustered into four groups that closely follow the four histological types. Clear cell tumors (ccRCC) clustered closer to the papillary tumors (pRCC) whereas renal oncocytoma (onco) and chromophobe tumors (chRCC) clustered together. (**B**) Classification scheme for renal cell carcinoma subtypes and renal oncocytoma. First, samples are classified into either ccRCC and pRCC or chRCC and oncocytoma using expression of miR221 and miR-222. In the second step, ccRCC is differentiated from pRCC by miR-126, miR-222 and miR-221, and chRCC is differentiated from oncocytoma using expression of miR-200b, miR-200c and miR-222. ccRCC = clear cell renal cell carcinoma; pRCC = papillary RCC; chRCC = chromophobe RCC.

In our previously published data [19], we developed a classifier that can distinguish major subtypes of kidney tumors by pairwise comparison of the expression of 40 miRNAs in fresh tissues in a maximum of four steps. In order to translate this discovery into the clinic using a smaller number of highly informative miRNAs that can be measured on FFPE tissues, we tested six miRNAs (miR-221, miR-222, miR-126, miR-182, miR-200b, and miR-200c) with potential utility as discriminatory markers. Selection criteria included frequency of dysregulation among subtypes, significance of differential expression, fold change, and evidence in literature of involvement in tumorigenesis. We quantified the expression of these miRNAs in ninety FFPE renal tumor samples by qRT-PCR. Five of these miRNAs showed differential expression between ccRCC, pRCC, chRCC and renal oncocytoma. Unsupervised clustering shows that with minimal exceptions, species of each histological type can be clustered together based on the expression of five specific miRNAs (Figure 1A).

### A miRNA classification system for distinguishing renal cell carcinoma subtypes

**Figure 2:**
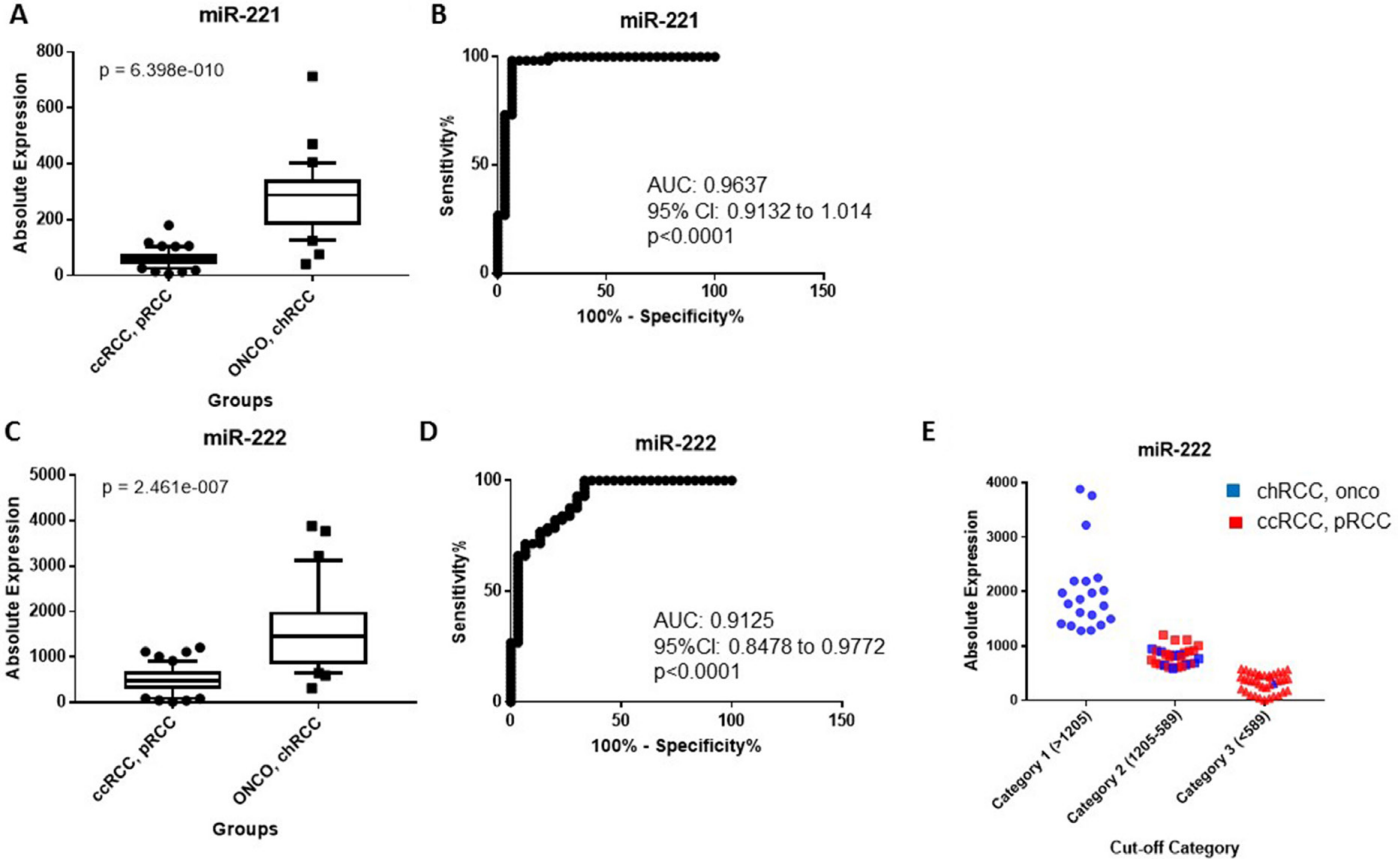
(**A**) miR-221 is significantly elevated in chRCC and renal oncocytoma relative to ccRCC and pRCC. (**B**) Receiver operating characteristic (ROC) curve showing the discriminatory ability of miR-221 to distinguish chRCC and renal oncocytoma from ccRCC and pRCC. (**C**) miR-222 is significantly overexpressed in chRCC and renal oncocytoma compared to ccRCC and pRCC. (**D**) ROC curve showing the discriminatory ability of miR-222 to distinguish the subtype pairs. Box plots (A and C) mean value (horizontal line), 10 to 90 percentile (box) and extent of data (whiskers). (**E**) Classification of tumors using a 3-point expression cut-off for miR-222. An expression cut-off was applied at >1205 copies (Category 1), between an expression range of 1205-589 copies (Category 2) and at < 589 copies (Category 3). ccRCC = clear cell renal cell carcinoma; pRCC = papillary RCC; chRCC = chromophobe RCC; onco = oncocytoma.

We developed a miRNA classification system able to differentiate the most common RCC subtypes and renal oncocytoma in two steps, as detailed in Figure 1B. In the first step, the expression of miR-221 and miR-222 were able to differentiate ccRCC and pRCC from chRCC and oncocytoma (Table 1). miR-221 was significantly upregulated in chRCC and oncocytoma compared to ccRCC and pRCC (4.49-fold change, *p* = 6.39 × 10^−10^) (Figure 2A), and was able to discriminate between the two groups (AUC: 0.96, 95% CI: 0.9132–1.014, *p* < 0.0001) (Figure 2B). miR-222 levels were significantly elevated in chRCC and oncocytoma (3.15-fold change, *p* = 2.46 × 10^−7^) (Figure 2C), and showed significant discriminatory ability between the subtype pairs (AUC: 0.91, 95% CI: 0.8478 to 0.9772, *p* < 0.0001) (Figure 2D). As outlined in Table 1, miR-221 correctly identified 83 of 86 cases (96.5%) whereas miR-222 accurately predicted 73 of 86 cases (84.9%). As illustrated in Figure 2C, expression of miR-222 did not allow for complete separation between the subtype pairs. Mislabeled tumors from both groups fell into an overlapping “grey zone”. In order to improve our biomarker performance, a 3-point cut-off was applied (Figure 2E). All cases were correctly identified as either chRCC or oncocytoma at a cut-off of > 1205 copies whereas 37 of 38 cases (97.4%) were correctly identified as either ccRCC or pRCC at a cut-off of < 589 copies. All misclassified cases, except one, fell within the range of 589-1205 copies. The discriminatory ability of miR-221 and miR-222 combined was less than miR-221 alone (AUC: 0.93, *p* < 0.0001).

**Table 1:** Expression of our miRNA classifiers in four histological types of renal tumors

	Mean expression				ccRCC + pRCC versus oncocytoma + chRCC				ccRCC versus pRCC				Oncocytoma versus chRCC			
miRNA	ccRCC	pRCC	Oncocytoma	chRCC	p-value	Fold	AUC	Accuracy (%)	p-value	Fold	AUC	Accuracy (%)	p-value	Fold	AUC	Accuracy (%)
miR-221	74.44	49.06	240.7	295.6	6.39 × 10−10	4.49	0.96	96.51	0.0013	1.52	0.75	69.6	0.26	1.23	0.63	70
miR-222	707.2	312.9	947.5	1951	2.46 × 10−7	3.15	0.91	84.88	3.47 × 10−8	2.26	0.88	78.57	0.0002	2.1	0.87	83.33
miR-126	3203	308	-	-	-	-	-	-	1.24 × 10−13	10.4	1	100	-	-	-	-
miR-200b	-	-	85.93	283.6	-	-	-	-	-	-	-	-	1.75 × 10−6	3.30	0.95	90
miR-200c	-	-	150	503.8	-	-	-	-	-	-	-	-	0.002	3.36	0.82	83.33

ccRCC = clear cell renal cell carcinoma; pRCC = papillary renal cell carcinoma; chRCC = chromophobe renal cell carcinoma.

Relative expression values.

**Figure 3:**
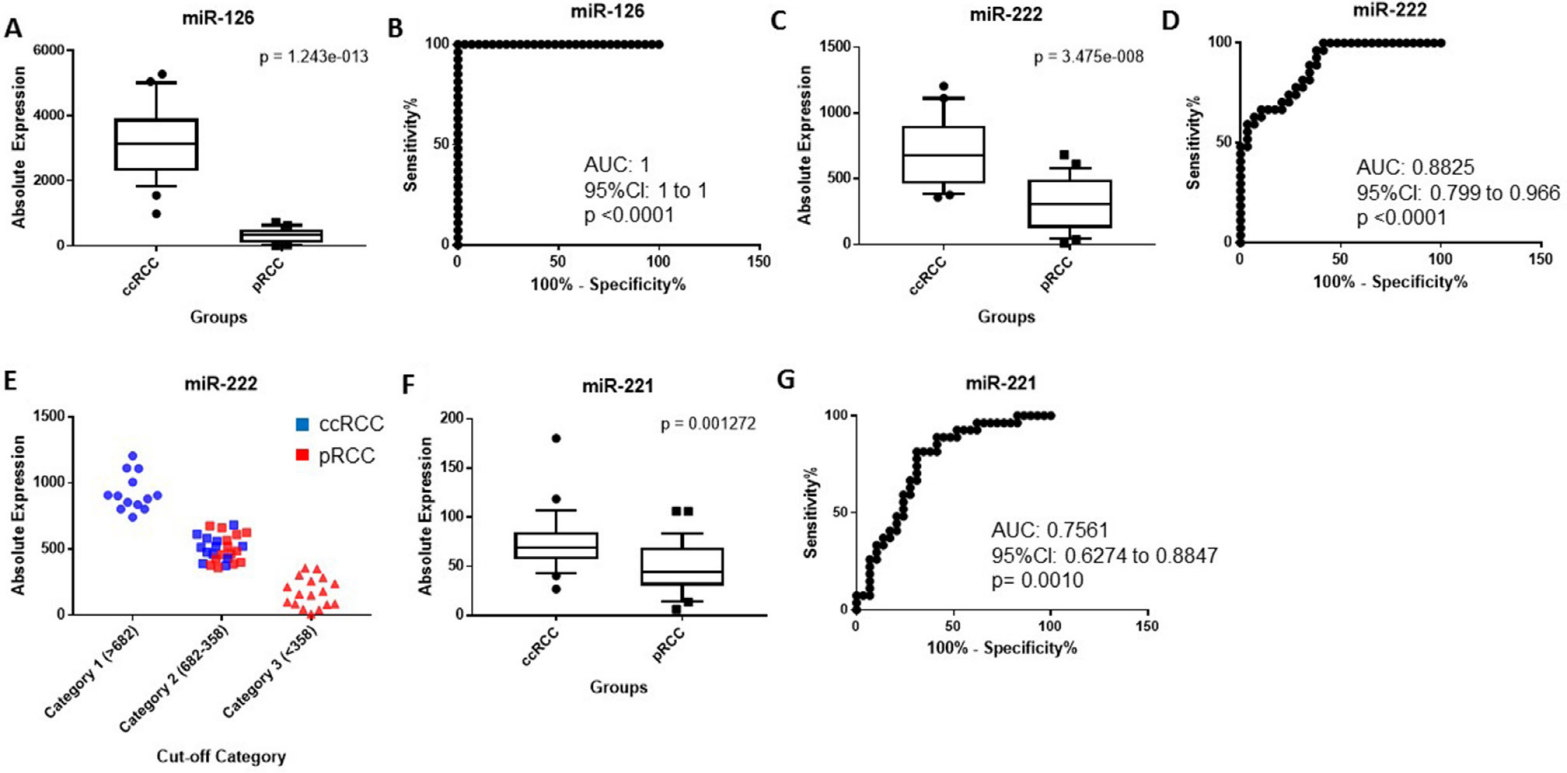
(**A**) miR-126 is significantly overexpressed in ccRCC compared to pRCC. (**B**) ROC curve showing the discriminatory ability of miR126 to discriminate ccRCC from pRCC. (**C**) miR-222 is significantly upregulated in ccRCC compared to pRCC. (**D**) ROC curve showing the discriminatory ability of miR-222 to distinguish the two groups. (**E**) Classification of ccRCC and pRCC using a 3-point expression cut-off for miR-222. An expression cut-off was applied at > 682 copes (Category 1), between an expression range of 682-358 copies (Category 2) and at < 358 copies (Category 3). (**F**) miR-221 is significantly elevated in ccRCC relative to pRCC. (**G**) ROC curve showing the discriminatory ability of miR-221 to discriminate ccRCC from pRCC. Box plots (A, C and E) mean value (horizontal line), 10 to 90 percentile (box) and extent of data (whiskers). ccRCC = clear cell renal cell carcinoma; pRCC = papillary RCC.

In the second step, the expression of miR-126, miR-182, miR-222 and miR-221 were tested to distinguish ccRCC from pRCC whereas miR-200b, miR-200c, miR-222 and miR-221 were tested to differentiate chRCC from oncocytoma (Table 1). miR-126 was significantly overexpressed in ccRCC compared to pRCC (10.4-fold change, *p* = 1.24 × 10^−13^) (Figure 3A), and was able to significantly discriminate between the two subtypes (AUC: 1, *p* < 0.0001). It correctly classified all 56 cases (100%) (Table 1, and Figure 3B). miR-222 was also significantly upregulated in ccRCC (2.26-fold change, *p* = 3.47 × 10^−8^) (Figure 3C), although some cases were still overlapping (AUC: 0.88, 95% CI: 0.799 to 0.966, *p* < 0.0001) (Figure 3D). When a 3-point cut-off was applied (Figure 3E), all cases were correctly identified as ccRCC at a cut-off of > 682 copies whereas all cases were accurately identified as pRCC at a cut-off of < 358 copies. Cases that were not accurately classified had expression levels between 682-358 copies. The expression of miR-221 was elevated in ccRCC relative to pRCC (1.51-fold change, *p* = 0.0012) (Figure 3F), and had much less discriminatory power (AUC: 0.75, 95% CI: 0.6274 to 0.8847, *p* = 0.001) (Figure 3G). miR-222 correctly predicted 44 of 56 cases (78.6%) whereas expression of miR-221 correctly classified 39 of 56 cases (69.6%) as either ccRCC or pRCC, as shown in Table 1. The expression of miR-182 could not differentiate ccRCC from pRCC (data not shown). The discriminatory ability of miR-221 and miR-222 combined was less than miR-126 alone (AUC: 0.88, 95% CI: 0.7954 to 0.9645, *p* < 0.0001).

**Figure 4:**
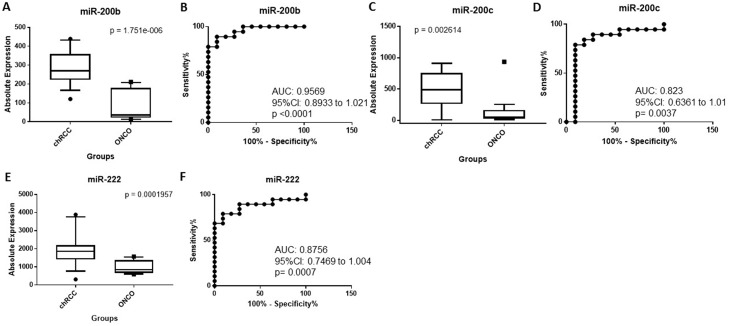
(**A**) miR-200b is significantly upregulated in chRCC compared to oncocytoma. (**B**) ROC curve showing the discriminatory ability of miR-200b to distinguish chRCC from oncocytoma. (**C**) miR-200c is significantly overexpressed in chRCC relative to oncocytoma. (**D**) ROC curve showing the discriminatory ability of miR-200c to distinguish chRCC from oncocytoma. (**E**) miR-222 is significantly elevated in chRCC relative to oncocytoma. (**F**) ROC curve showing the discriminatory ability of miR-222 to discriminate chRCC from oncocytoma. Box plots (A, C and E) mean value (horizontal line), 10 to 90 percentile (box) and extent of data (whiskers). chRCC = chromophobe RCC; onco = oncocytoma.

The absolute expression of miR-200b was significantly higher in chRCC relative to oncocytoma (3.30-fold change, *p* = 1.75 × 10^−6^) (Table 1 and Figure 4A). As illustrated in Figure 4B, miR-200b was able to distinguish chRCC from renal oncocytoma (AUC: 0.95, 95% CI: 0.8933 to 1.021, *p* < 0.0001) with a sensitivity of 89.5% and a specificity of 90.9% at a cut-off of 197 copies. miR-200b correctly identified 27 of 30 cases (90%) as either chRCC or renal oncocytoma. miR-200c was significantly upregulated in chRCC (3.36-fold change, *p* = 0.0026) (Table 1 and Figure 4C), and was able to discriminate between the two subtypes (AUC: 0.82, 95% CI: 0.6361 to 1.01, *p* = 0.0037) with 84 % sensitivity and 82 % specificity at a cut-off 180 copies (Figure 4D). It correctly classified 25 of 30 cases (83%). miR-222 was also upregulated in chRCC compared to renal oncocytoma although to a lesser degree (2.1-fold change, *p* = 0.0002) (Figure 4E), and had a lower sensitivity relative to miR-200b and miR-200c (AUC: 0.87, 79 % sensitivity and 91% specificity at a cut-off 1399 copies) (Figure 4F). miR-222 accurately classified 25 of 30 cases (83%). Although miR-221 was elevated in chRCC relative to renal oncocytoma, this did not reach statistical significance (Table 1). The discriminatory ability of miR-200b and miR-200c combined was less than miR-200b alone. This was also seen for the combination of miR-222 and miR-200b or miR-200c (data not shown).

### miRNA expression can help stratify unclassified RCC

We tested the ability of miR-221, miR-222, miR-126, miR-200b and miR-200c, which were included in the classification scheme, to stratify unclassified RCC. Sample A had extensive necrosis with few viable areas. Microscopic and immunohistochemical evaluation was inconclusive as the tumor displayed overlapping morphology of both clear cell and papillary subtypes (Supplementary Figure 2). Sample B displayed overlapping morphology of an eosinophilic RCC. Immunostaining was non-contributory. Sample C displayed variable morphologic types of renal cell carcinoma with areas of sarcomatoid differentiation, and sample D had mixed morphology.

As illustrated in Supplementary Figure 3A–3B), samples A, C and D classified as ccRCC or pRCC by miR-221 and miR-222 expression whereas sample B classified as chRCC or renal oncocytoma. Sample A and C were favoured to be pRCC whereas Sample D classified as ccRCC by miR-126 expression, as seen in Supplementary Figure 3C. Sample A was further confirmed as pRCC by miR-222 expression whereas samples C and D were inconclusive because their expression fell within the grey zone of miR-222 expression (between 682-358 copies, Supplementary Figure 3D). Samples A, C and D could not be accurately classified by miR-221 expression (data not shown). Sample B was further suggested to be renal oncocytoma using both miR-200b and miR-200c expression, as seen in Supplementary Figure 3E–3F), respectively, while using miR-222 expression, it was favoured to be chRCC (Supplementary Figure 3G). The above findings suggest that our miRNA classifier can be used to supplement morphology in defining the nature of the tumor, and also highlight the difficulty in separating chRCC from oncocytoma as discrete entities.

### miRNA *in situ* hybridization in renal cell carcinoma subtypes

**Figure 5:**
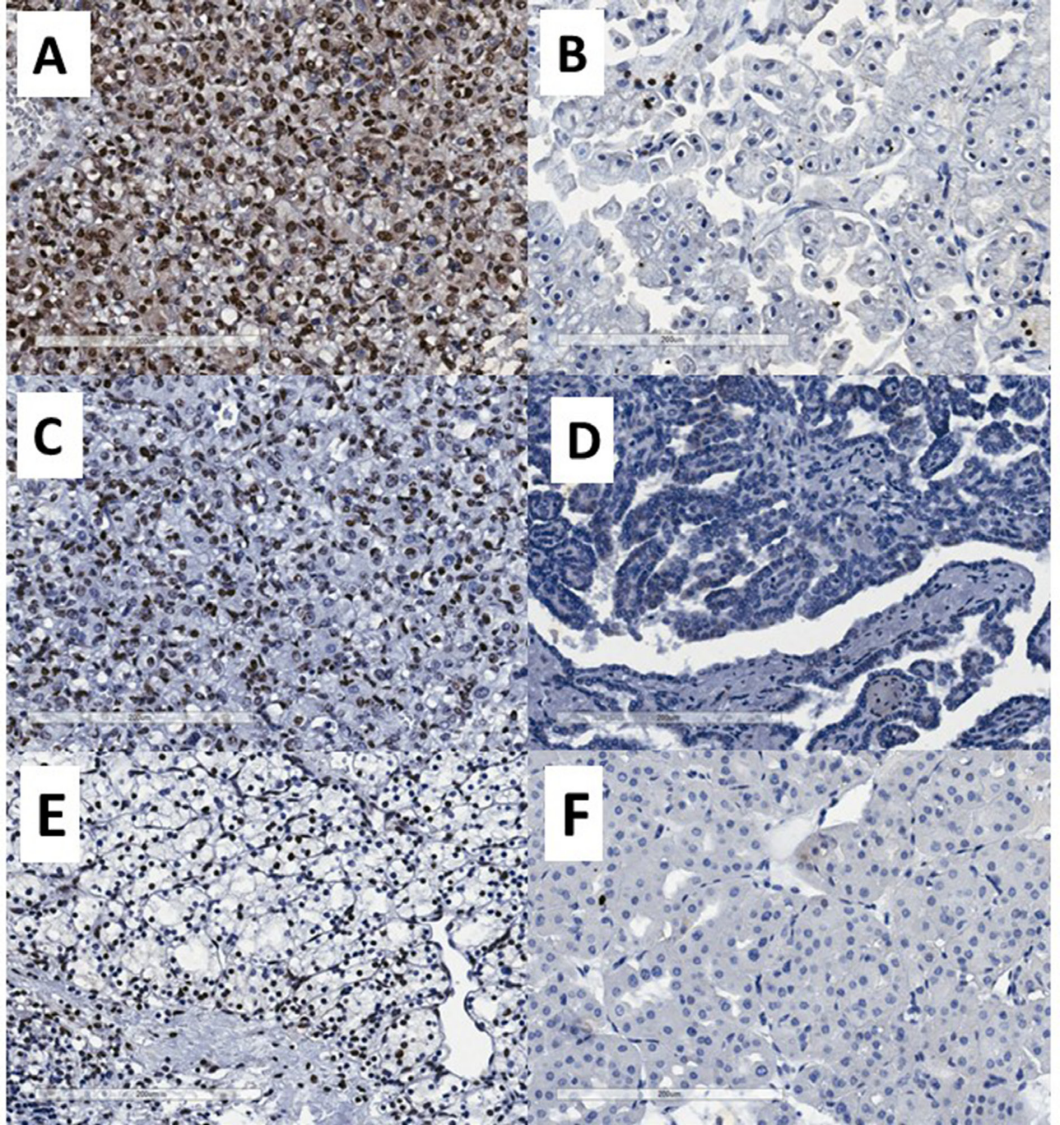
Representative photomicrographs showing miRNA expression by chromogenic *in situ* **hybridization.** (**A**) strong miR-126 nuclear expression, (**B**) weak/negative miR-126 nuclear expression, (**C**) strong miR-222 nuclear expression, (**D**) negative miR-222 nuclear expression, (**E**) strong miR-200b nuclear expression and (**F**) negative miR-200b nuclear expression.

We next examined the expression of miR-126, miR-222 and miR-221, assessed through in-situ hybridization, can be valuable markers to differentiate between the three most common RCC subtypes and oncocytoma. Representative stating patterns are shown in Figure 5. Using nuclear expression of miR-126 as a dichotomous variable, 89% of ccRCC tumors were positive compared to only 11% of pRCC (*p* < 0.0001) (Table 2). The same pattern was seen for miR-222, with 71% of ccRCC tumors positive compared to only 29% of pRCC tumors showing positive expression (*p* = 0.013). A similar trend was seen for nuclear miR-221 expression, however this did not reach statistical significance (*p* = 0.0536).

**Table 2:** Associations between nuclear miR-126, miR-222 and miR-221 expression, as dichotomous variable, and RCC patient diagnosis

Variable	total	No. of patients (%)		*p*-value
		negative	positive	
**miR-126**				
pRCC	28	24 (85.71)	4 (10.81)	< 0.0001
ccRCC	37	4 (14.29)	33 (89.19)	
**miR-222**				
pRCC	28	18 (60.00)	10 (28.57)	0.0134
ccRCC	37	12 (40.00)	25 (71.43)	
**miR-221**				
pRCC	28	12 (63.16)	16 (34.78	0.0536
ccRCC	37	7 (36.84)	30 (65.22)	

ccRCC = clear cell renal cell carcinoma; pRCC = papillary RCC

Bold refers to statistically significant values

Calculated using Fisher's Exact test.

In addition, we assessed nuclear expression of miR-200b, miR-200c and miR-222 as a dichotomous variable in chRCC and oncocytoma. miR-200b was positive in 92% of chRCC compared to only 8% oncocytoma cases (*p* = 0.009) (Table 3). A similar trend was seen for nuclear expression of miR-200C, with 73% positivity in chRCC compared to only 27% of oncocytoma cases, although this was not statistically significant (*p* = 0.064) (Table 3). Nuclear miR-222 expression did not show statistically significant differences.

**Table 3:** Associations between nuclear miR-200b, miR-200c and miR-222 expression, as dichotomous variable, and RCC patient diagnosis

Variable	total	No. of patients (%)		*p*-value
		negative	positive	
**miR-200b**				
chRCC	20	9 (42.86)	11 (91.67)	**0.009**
Oncocytoma	13	12 (57.14)	1 (8.33)	
**miR-200c**				
chRCC	20	4 (36.36)	16 (72.73)	0.0645
Oncocytoma	13	7 (63.64)	6 (27.27)	
**miR-222**				
chRCC	20	5 (71.43)	15 (57.69)	0.6756
Oncocytoma	13	2 (28.57)	11 (42.31)	

chRCC = chromophobe RCC; onco = oncocytoma

Bold refers to statistically significant values

Calculated using Fisher's Exact test.

## DISCUSSION

RCC represents a heterogeneous disease that encompasses a spectrum of distinct subtypes. The ability to subclassify RCC is of clinical importance since each subtype is a genetically distinct tumor with varying clinical course, prognosis and potential response to treatment. Currently, routine subtype classification relies on H&E morphology and immunohistochemistry , however this is not always conclusive due to inter-observer variability and overlapping morphology [6].

miRNAs, a class of small non-coding RNAs have gained recognition as promising biomarkers [22]. The specificity and stability of miRNAs in FFPE tissues has encouraged their use as markers to subclassify RCC [23]. In the current study, we investigated the utility of miRNAs as potential biomarkers to aid in classifying RCC. We developed a miRNA classification system that can distinguish between the most common RCC subtypes and renal oncocytoma in two steps based on the expression of five miRNAs. We found that miR-221 and miR-222 could significantly distinguish ccRCC and pRCC from chRCC and oncocytoma. This is consistent with previous studies that reported elevated miR-221 levels in chRCC and oncocytoma relative to ccRCC and pRCC [20]. Clear cell and papillary RCC are believed to arise from the proximal tubules, while chRCC and oncocytoma are from the distal tubules [24]. Our results align with this since miR-221 and miR-222 expression separated ccRCC and pRCC from chRCC and oncocytoma. miR-126, miR-222 and miR-221 were able to significantly differentiate between ccRCC and pRCC. Several studies have reported elevated expression of miR-126 in ccRCC compared to pRCC [20, 25]. miR-126 is commonly associated with angiogenesis [26], which is a much more prominent feature ccRCC, compared to pRCC [27].

Expression levels of miR-200b, miR-200c and miR-222 were found to significantly differentiate chRCC from renal oncocytoma, although miR-200b had better discriminating ability. This is consistent with several reports that found miR-200b [20, 25] and miR-200c [20, 28] to be significantly elevated in chRCC compared to oncocytoma. Of the miRNAs included in our classification system, miR-221 had the best diagnostic performance in discriminating ccRCC and pRCC from chRCC and renal oncocytoma. miR-126 and miR-200b had the best diagnostic performance in discriminating ccRCC from pRCC and chRCC from renal oncocytoma, respectively. Upon review of cases that were incorrectly predicted, some had mixed morphology and were re-classified as hybrid oncocytic chromophobe tumors (HOCT). Our study is in keeping with the recent trend suggesting that oncocytoma and chRCC occupy opposite ends of the same tumor continuum. The use of histology as the gold standard for classification represents a limitation for our study.

RCC subtypes exhibit characteristic chromosomal aberrations, such as whole or partial chromosomal duplications and deletions. Chromosomal alterations might explain, at least in part, the differential miRNA expression pattern observed among RCC subtypes. miR-221 and miR-222 are closely located on chromosome Xp11.3 [29]. Loss of chromosome X was previously reported in pRCC [30]. This is consistent with our results, since miR-222 expression was significantly lower in pRCC relative to ccRCC. miR-126 is located in the intronic region of the epidermal growth factor-like 7 gene (EGFL7) on chromosome 9 [31]. Frequent loss of chromosome 9p was also previously reported in pRCC [32], which may explain why expression levels of miR-126 are decreased in pRCC relative to ccRCC. Interestingly, loss of chromosome 9p and 9q have also been reported in ccRCC [32], however this occurs at a later stage of disease progression [33].

Our study emphasizes the recent trend towards “molecular” classification of RCC. Brannon et al. identified two distinct ccRCC subtypes, designated clear cell type A (ccA) and B (ccB) [34]. Papillary tumors can also be subdivided into at least two subtypes, type I and II, based on distinct microscopic and gene expression patterns [35]. Oncocytic low-grade pRCC has also been described [36, 37]. These subtypes likely display distinct cytogenetic aberrations. For instance, chromosome 1p, 3p and 9p loss in addition to frequent gain of 5q and 8q are exclusively seen in type II [10] relative to papillary type I [13]. Oncocytic low-grade pRCC has chromosomal gains closer to type I pRCC [38], although more work is needed to assess the global cytogenetic aberrations of this tumor. Moreover, variants of chRCC exist, including an eosinophilic variant [39]. These variants may exhibit distinct molecular signatures. In fact, although classic chromophobe tumors show frequent loss of chromosomes 1, 2, 6, 8,10, 13, 17 and 21, the eosinophilic variant was found to be mostly diploid [40].

miR-200b maps to chromosome 1p36.33 [21]. Several studies have reported high prevalence of chromosome 1 or 1p loss in renal oncocytoma [21, 41] , which would explain the lower miR-200b expression levels in oncocytoma. Moreover, miR-200c is located on chromosome 12 p13.31 [21]. Frequent copy number gains in chromosome 12 have been reported in chRCC [42]. Again, this is consistent with our results showing elevated miR-200c in chRCC.

Interestingly, select miRNAs were useful in pointing to distinct histological types in unclassified RCC, as seen above. Classification of RCC tumors can present a significant diagnostic challenge in cases where tumors display overlapping features [43]. Moreover, inter-observer variability amongst pathologists can also make the differential diagnosis of tumors challenging [6]. Our results show a potential benefit of miRNA-based *in situ* hybridization assay using miRNA-specific probes in FFPE tissues to aid in the classification of common RCC subtypes. This can complement routine hematoxylin-eosin staining for improved RCC diagnosis. More detailed analysis of less-common subtypes are needed to further validate the performance of our markers.

We do acknowledge some limitations of our study. Independent validation is needed on larger case numbers. Also, we only focused on cases with classic morphology. We found that *in situ* hybridization showed less ability to accurately quantify miRNA expression levels compared to qRT-PCR analysis. Previous studies have shown that automated imaging algorithms are able to quantify staining intensity with high sensitivity [44, 45]. Thus, pairing chromogenic *in situ* hybridization to an automated imaging algorithm may increase the sensitivity of this platform.

A frequent struggle for pathologists is the accurate classification of incidentally detected small renal masses (pT1a, ≤ 4 cm) on renal biopsy. Here, diagnostic material is often limited and morphology is in some cases inconclusive [46]. We speculate that our miRNA classifier may be useful for better classification of these cases on needle core biopsy. This would have significant implications for treatment of renal oncocytoma ≤ 4 cm, where active surveillance may be considered as an alternative option to immediate intervention [47]. In the clinical setting, qRT-PCR is feasible. It is possible to obtain ample amounts of RNA (0.5–1.3 μg/20 sections) from needle core biopsy [48]. Moreover, the qRT-PCR platform is also amenable to multiplexing which allows for quick turnaround. As mentioned above, chromogenic *in situ* hybridization can also be incorporated into routine hematoxylin-eosin staining of needle core biopsies for improved classification of early-stage tumors.

Herein we demonstrate that the expression levels of a limited panel of miRNAs, including miR-221, miR-222, miR-126, miR-200b and miR-200c can help in subclassifying the most common RCC subtypes as well as renal oncocytoma. In addition, these miRNAs can provide insight into the molecular characteristics of individual unclassified tumors which may help in the selection of effective targeted therapies. Importantly, the miRNA classifier was validated by *in situ* hybridization, demonstrating the feasibility of this platform in classifying RCC. To our knowledge, we are the first to assess the staining patterns of miR-221, miR-222, miR-126, miR-200b and miR-200c by *in situ* hybridization for the differential diagnosis of RCC.

## MATERIALS AND METHODS

### Specimen collection for RNA extraction

For PCR analysis, we collected a total of 90 formalin-fixed paraffin-embedded RCC tissues from the archives of the department of laboratory medicine at St. Michael's Hospital, Canada from 2002–2012 for qRT-PCR analysis. Diagnosis was reviewed according to the most recent Vancouver ISUP classification [4]. These included 27 ccRCC cases, 29 pRCC cases, 19 chRCC cases, 4 unclassified RCC cases and 11 oncocytoma cases. miRNA *in situ* hybridization was done on in an independent set of 98 FFPE RCC tissues.

### RNA extraction

Sections of the formalin-fixed paraffin-embedded tissue corresponding to the designated subtype were selected for RNA extraction. Six cores of pure tumor tissue were obtained and pooled for each specimen to compensate for tumor heterogeneity. Tissues were taken from areas with no hemorrhage or necrosis. Total RNA was extracted using the miRNeasy FFPE Kit (Qiagen, Mississauga, Ontario) according to the manufacturer's protocol. RNA quality and concentration were determined spectrophotometrically (NanoDrop 2000c spectrophotometer, NanoDrop Technologies Inc., Wilmington, DE). Optimal RNA samples were stored at -80°C.

### miRNA quantification

miRNA-specific reverse transcription was performed with 350 ng total RNA using the TaqMan microRNA Reverse Transcription Kit (Applied Biosystems, Foster City, CA) as recommended by the manufacturer. RT-qPCR was performed using the TaqMan microRNA Assay Kit on Viia7 Real-Time PCR System (Applied Biosystems) [49] using miRNA-specific primers and probes. Thermal cycling conditions were according to the manufacturer's standard protocol and all reactions were performed in triplicates (Supplementary Figure 4). For generation of standard curves of chemically synthesized RNA oligonucleotides corresponding to miRNAs of interest, serial dilutions of each oligonucleotide were made in nuclease free water such that the final input into the RT reaction had a volume of 5 uL. Copy number was calculated using EndMemo (http://www.endmemo.com/bio/dnacopynum.php). A line was fit to the data from each dilution series using Ct values within the linear range, from which y = mln(×) +b equations were derived for quantification of absolute miRNA copies (×) from each sample Ct (y). A representative standard curve is illustrated in Supplementary Figure 1.

### Tissue microarray construction

Tissue microarrays (TMAs) were built from 98 RCC formalin fixed paraffin-embedded tissue blocks from the surgical pathology archives of St. Michael's Hospital between 2001-2009. Diagnosis was confirmed by a pathologist according to the recent ISUP 2012 classification. These included 37 ccRCC cases, 28 pRCC cases, 20 chRCC cases and 13 oncocytoma cases. Each specimen was represented by two 1 mm cores obtained from two separate blocks to account for tumor heterogeneity. Areas of necrosis were avoided. Paraffin sections of the TMA were cut in 4 um thickness for *in situ* hybridization. The study was approved by the Research Ethics Board from St. Michael's Hospital, Toronto, Canada.

### *In situ* hybridization

miRNA expression was assessed in FFPE TMA samples by *in situ* hybridization (ISH) performed by BioGenex Laboratories Inc. (Fremont, CA) with miRNA ISH probes and Super Sensitive One-step Polymer-HRP ISH detection kit on Xmatrx, the fully automated staining system (BioGenex Laboratories). In brief, these tissues were pre-treated with nucleic acid retrieval (NAR) solution at 85°C for 5 minutes and 100°C for 20 minutes. Then the tissues were incubated with hybridization buffer at 42°C for 20 minutes, followed by ISH using Fluorescein (FAM)-labeled miRNA probes for miR-200b, miR-200c, miR-126 and miR-222 (100 nM) at 42°C for 2 hours. The non-specific binding probes were removed by stringent washes at 42°C. The probes were detected by sequential addition of anti-fluorescein antibody followed by poly-HRP. Final color visualization was performed by DAB along with hematoxylin counter staining.

### *In situ* hybridization scoring

Hybridization signals were assessed semi-quantitatively by two of the authors (A.D., R.S.)using a combined score resulting from summing the intensity of staining and frequency of immunoreactive cells. Staining intensity was 4-tier, including 0 (negative), 1 (weak), 2 (moderate), and 3 (strong). Frequency of immunoreactive cells was ranked in a 4-tier, including 0 (0%), 1 (< 33%), 2 (34% to 66%) and 3 (> 66%). Scores for miRNA staining were dichotomized into two groups; negative (0 to 2) and positive (3 to 6).

### Statistical analysis and clustering

GraphPad Prism 7.1 and Perseus were used for statistical analysis. RT-PCR measurements (*C*_t_ values) for 6 miRNAs were obtained for each specimen. *C*_t_ values greater than 35 were considered to be non-expressed and truncated to 35. The unknown absolute expression level of each miRNA was interpolated from a semi-log curve. For every group (e.g. Oncocytoma versus chromophobe or clear cell versus papillary), absolute miRNA expression was compared for all miRNAs. The discriminatory power of miRNA combinations, calculated as a sum of absolute miRNA expression was also determined. *P* values were calculated using a Welch's two-tailed Student's *t*-test. For differentially expressed miRNAs receiver operator characteristic (ROC) curves were constructed and the areas under the curve (AUC) were calculated to assess diagnostic performance. For *in situ* hybridization, Fisher's Exact Test was used to evaluate associations between miRNA staining pattern and RCC diagnosis. The Wilson-Brown method was used to compute confidence intervals (CI’s), sensitivity and specificity. Hierarchical clustering was performed using GenePattern software (https://genepattern.broadinstitute.org, accessed November, 2016) [50, 51]. Absolute expression values were log-transformed and Pearson correlation was used to calculate the distance between every pair of samples.

## SUPPLEMENTARY MATERIALS FIGURES



## Abbreviations

ccRCC: clear cell renal cell carcinoma
pRCC: papillary RCC
chRCC: chromophobe RCC
HOCT: hybrid oncocytic chromophobe tumors
ISH: *in situ* hybridization
