# Supplementary material for: A miRNA-based classification of renal cell carcinoma subtypes by PCR and in situ hybridization
Source: Oncotarget. 2017 Dec 8;9(2):2092–104. doi: 10.18632/oncotarget.23162 (PMC5788624; doi:10.18632/oncotarget.23162)
Supplement: Supplementary file 1 [file oncotarget-09-2092-s001.pdf]

A miRNA-based classification of renal cell carcinoma subtypes by PCR and *in situ* hybridization

SUPPLEMENTARY MATERIALS

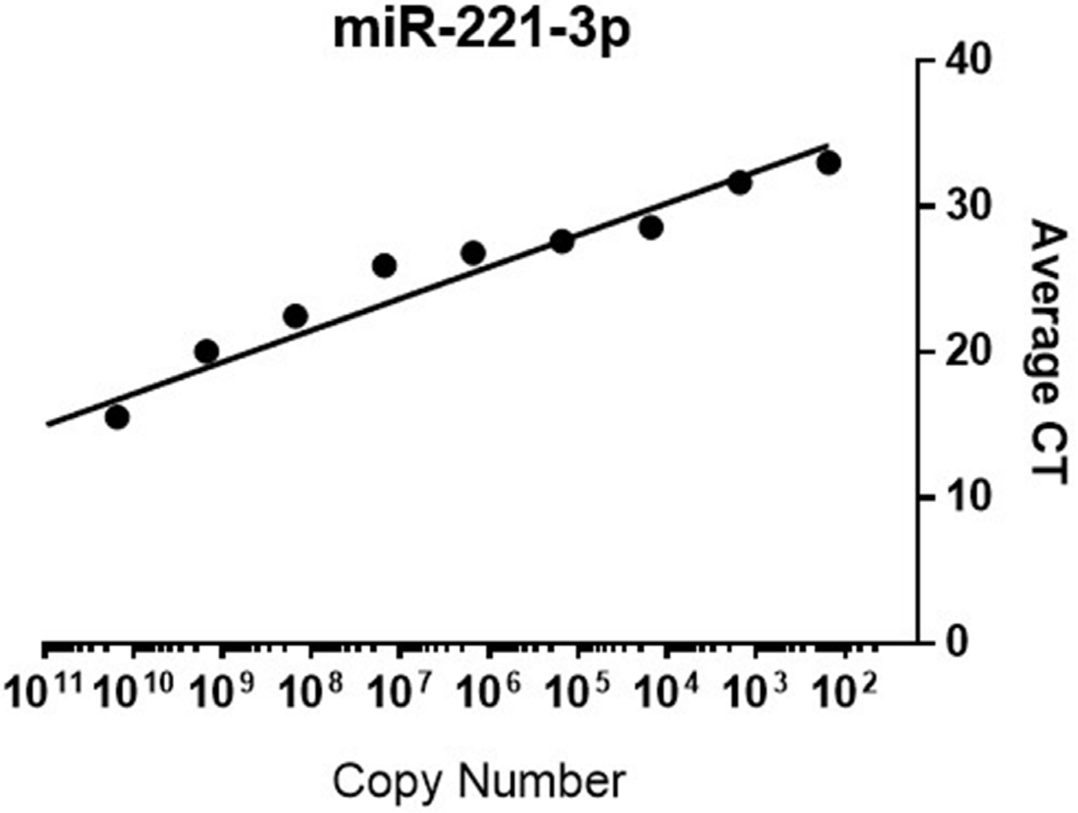

Supplementary Figure 1: Representative standard curve of miR-221-3p. The standard curve was generated using serial dilutions of known input amount of synthetic miR-221 oligonucleotide.

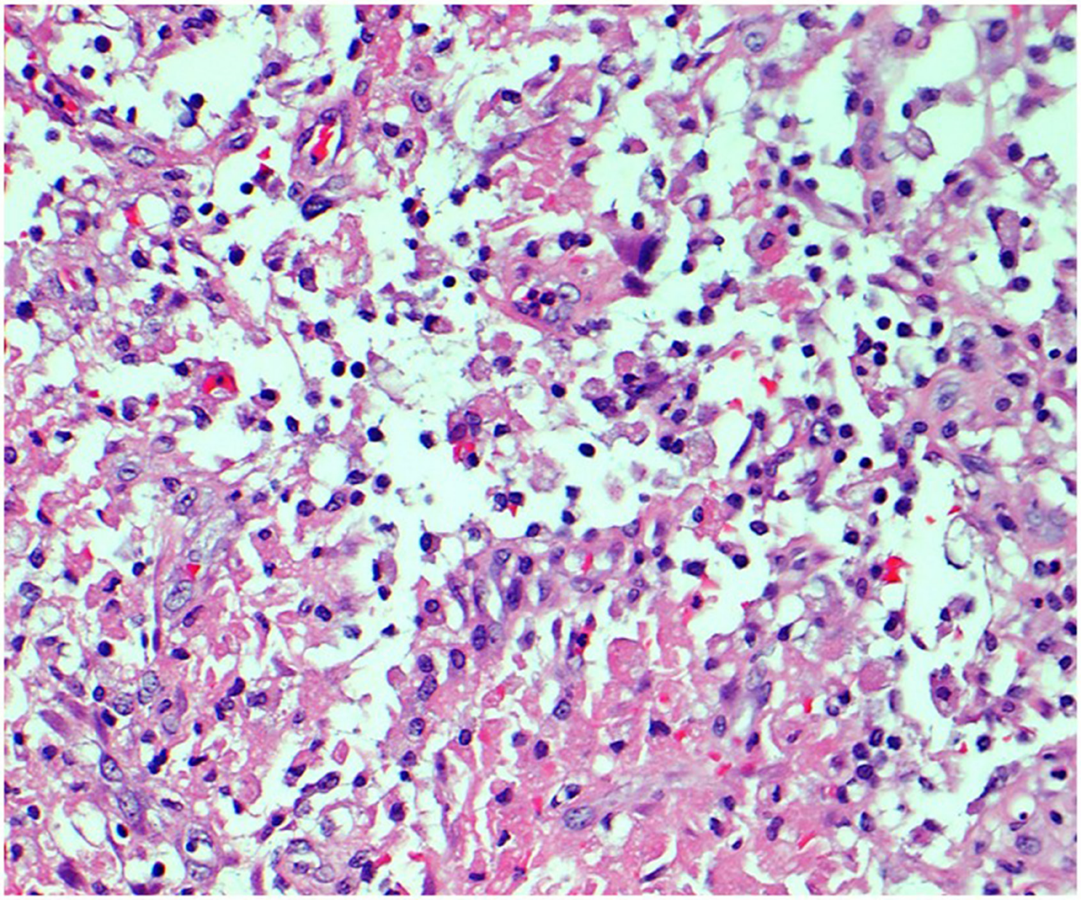

Supplementary Figure 2: Photomicrograph of unclassified RCC (Sample A). Note microscopic evaluation was inconclusive, tumor displayed overlapping morphology of both clear cell and papillary subtypes.

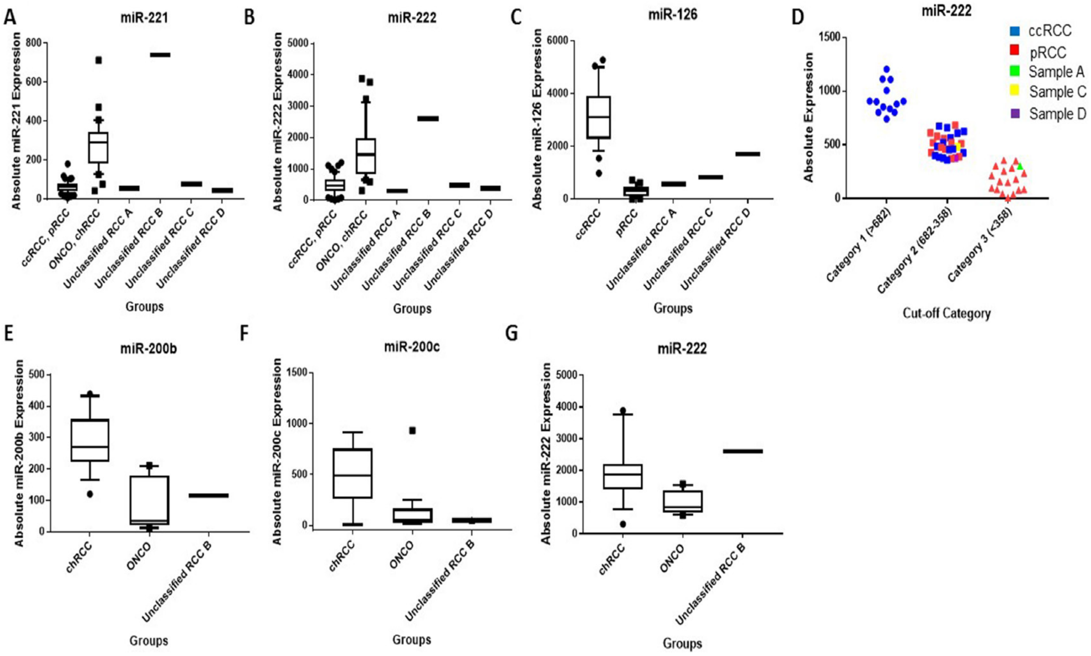

Supplementary Figure 3: miRNA classifier expression in cases of unclassified RCC. Absolute expressions of miR-221 (**A**), miR-222 (**B**), and miR-126 (**C**) were assessed in ccRCC, pRCC, chRCC, renal oncocytoma and 4 unclassified RCC cases (cases A-D). (**D**) Scatter plot showing miR-222 expression in unclassified RCC cases. Sample A fell below 358 copies (Category 3) whereas samples C and D fell in the intermediate expression range of 682-358 copies (Category 2). (**E**) Expression of miR-200b in chRCC, renal oncocytoma and unclassified RCC case B. (**F**) Expression of miR-200c in chRCC, renal oncocytoma and unclassified RCC case B. (**G**) Expression of miR-222 in chRCC, renal oncocytoma and unclassified RCC sample B. ccRCC = clear cell renal cell carcinoma; pRCC = papillary RCC; chRCC = chromophobe RCC; onco = oncocytoma.

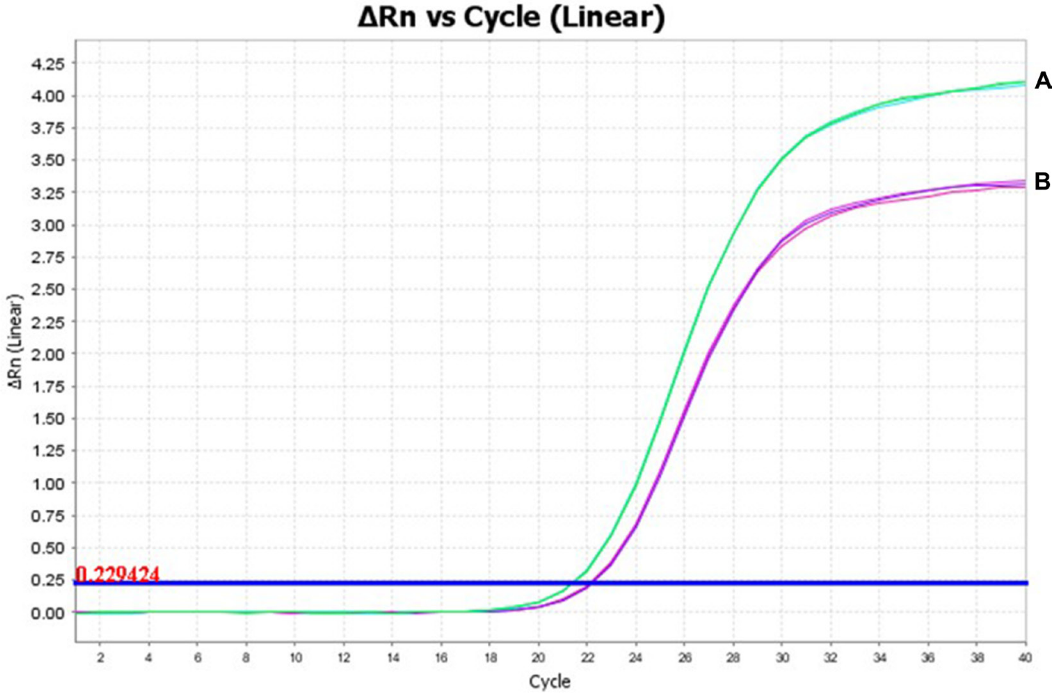

Supplementary Figure 4: Representative real-time PCR amplification plots for two RCC tissue samples. The curves represent technical replicates (in triplicate) of real-time PCR analysis for two RCC tissue samples A and B. The horizontal line represents the threshold, which was set automatically by the instrument.
